# Using mHealth to Support Queensland Mothers and Children From Birth to Two Years: A Longitudinal Study of Connecting2u

**DOI:** 10.1002/hpja.70117

**Published:** 2025-10-14

**Authors:** Lisa Irene Jones, Brett Dyer, Amanda Wedemeyer, Lieke Vorage, Nicola Wiseman, Alanna Philipson, Skye Frazer‐Ryan, Andrew Resetti, Neil Harris

**Affiliations:** ^1^ Griffith Health Griffith University Gold Coast Queensland Australia; ^2^ Children's Health Queensland Centre for Children's Health and Wellbeing South Brisbane Queensland Australia; ^3^ Reform Office Strategy Policy and Reform Division, Queensland Health Brisbane Queensland Australia

**Keywords:** child health, health promotion, infant, mHealth, mothers, parents, postpartum period

## Abstract

**Introduction:**

Connecting2u (C2u) is a mobile health (mHealth) intervention supporting Queensland families. The 2021–2023 iteration focused on the first 2 years of a child's life. This study evaluated whether C2u supported maternal and child well‐being during this period.

**Methods:**

This longitudinal study used a self‐administered online survey at four points over 2 years. Survey items included the Karitane Parenting Confidence Scale, Modified Medical Outcome Social Support survey, child immunisation status, agreement statements, and open‐ended questions on C2u support and satisfaction. Parenting confidence and social support were modelled using linear quantile mixed models; univariate and qualitative content analysis were applied to remaining items.

**Results:**

Parenting confidence increased over the first 12 months, with faster improvements among first‐time parents and modestly faster gains among parents with university‐level education. Social support scores showed little change over the 2‐year period, although qualitative data suggested C2u encouraged conversations between parents. Agreement statements indicated that C2u was well received and supported preventative health behaviours. Qualitative feedback reinforced these findings, with participants describing C2u as an extra layer of support, prompting self‐care, encouraging bonding with their child, and offering timely guidance on introducing solids. By the end of the study, 100% of participants indicated their child had received the full schedule of age‐appropriate immunisations.

**Conclusions:**

C2u was well received by mothers. Enhancing its focus on partner support, expanding social support networks, guidance on sibling bonding, and self‐care strategies could be beneficial. Queensland Health could consider implementing an online booking system to streamline child health appointments. So what? C2u is an innovative mHealth intervention. Our findings support refinement as C2u expands to include antenatal care messaging and support for children up to 5 years, helping to optimise outcomes for families.

## 1 Introduction

Early childhood is a critical period for a child's overall health and future health outcomes [1]. In particular, the first 1000 days of life (conception through age 24 months) are essential to cognitive, emotional, physical and physiological development [2]. Importantly, research highlights that early relational and bonding experiences after birth play a critical role in shaping mental health, emotional regulation, and social connectedness across the life course [3, 4]. These experiences can also influence overall well‐being more broadly, with established links between early childhood and the later development of chronic conditions such as obesity and type 2 diabetes [5, 6, 7]. Parents and caregivers therefore play a vital role in achieving optimal outcomes for their children. However, learning to parent an infant is a challenging transitional period, and it can significantly impact parents' own well‐being [8, 9, 10].

Parenting confidence, defined as a parent's sense of competence or self‐efficacy in their role [11], is linked to successful parenting practices and better health outcomes for both parents and children [8, 11]. For example, a mother's ability to care for her infant is closely associated with the child's cognitive, physical and emotional development. However, when a mother lacks confidence, this negatively influences their motherhood experience and therefore, their ability to care for their infant [12, 13, 14]. The literature suggests that receiving social support plays a crucial role in boosting maternal self‐confidence [15, 16], with social support being a strong predictor of maternal parental self‐efficacy [17].

Certainly, encouraging mothers to feel a part of a supportive network is important for promoting their physical, mental and emotional well‐being, particularly in the postpartum period [18]. A systematic review by Ni and Siew Lin [19], for instance, found that informal support from family and friends significantly reduces stress and postnatal depression. Additionally, increasing social support for postpartum women promotes breastfeeding and improves infant care. Social support can also protect against weak infant attachment [20], noting that maternal–infant bonding is essential for the psychological well‐being of the mother, and a child's survival and health outcomes [21, 22]. Separate research supports the need for social support as infants progress into their toddler years, with better relationship quality between couples a predictor for greater parental engagement with their children [23].

Another important factor in ensuring optimal health outcomes for children is the mother's own health [8, 24]. Based on an Australian birth cohort study, findings suggest that poor general health in mothers during the first year after childbirth is associated with higher odds of poor health in infants and adolescents [25]. Encouraging self‐care, including taking time for oneself, is crucial for improving both maternal and child well‐being [26]. In particular, formal social support, such as guidance from healthcare providers, plays an important role in supporting well‐being of mothers [27].

Healthcare providers are also important for providing guidance on feeding practices and carrying out child health appointments and vaccinations. Ensuring adequate nutrition during the first years of life is critical for physical and cognitive development, as well as for protecting against chronic diseases such as obesity [7, 28]. Likewise, ‘child health checks’ and vaccinations are important in preventative health efforts and ensuring healthy development of children [29, 30].

As low parental health literacy can greatly affect the ability to act upon health information and make health decisions for their child [31], it is important parents receive accurate information they are able to understand and use during the early years. There is a large amount of available information about parenting, with parents often reporting feeling overwhelmed by inconsistent and confusing information about effective parenting practices [32]. In addition, confidence in professional advice from health professionals can be eroded by conflicting information [33], indicating the need for consistent and accurate information disseminated by a trustworthy source. One dissemination method is the use of text messaging or short message service (SMS), with research showing this method to be an effective tool to simplify delivery of health information [34, 35].

As detailed by Children's Health Queensland [36] (CHQ), Connecting2u (C2u) is a health promotion intervention implemented by the Children's Health Queensland Hospital and Health Service (CHQ HHS) that utilises mobile health (mHealth) technology through SMS messages. While C2u has now been expanded to include antenatal care and children aged 0–5 years, between 2021 and 2023, C2u focused on supporting the well‐being of Queensland families during the first 2 years of their child's life (0–24 months). The messages, typically consisting of one to two sentences written from the baby's point of view, included information on child development, milestones, vaccination and other appointment reminders, breastfeeding, parents' self‐care, and psychosocial well‐being. C2u has four main targeted messaging streams catering for: mothers/carers, Aboriginal and/or Torres Strait Islander mothers/carers, fathers/carers, and Aboriginal and/or Torres Strait Islander fathers/carers.

The overarching aim of the 2021–2023 iteration of C2u was to equip parents with important skills, knowledge and resources to enhance parenting practices and parental health behaviours during their child's first 24 months. This study focused on those enrolled within the mothers' stream. It aimed to evaluate whether C2u supported the well‐being of mothers' and children, through the following objectives:

1. Empower mothers to maximise healthy development of their child through improving parental confidence,
2. Support positive relationships between mothers, their children and partner (where applicable); to make mothers feel part of a supportive network,
3. Support preventative health efforts including,
  - Supporting mothers' overall well‐being,
  - Encouraging positive feeding practices,
  - Promoting engagement with health services (including child health and vaccination appointments).
4. Explore overall satisfaction of C2u, including areas of improvement.

## 2 Methodology

### 2.1 Study Design

A longitudinal study including a self‐administered online survey was conducted with participants enrolled into the mothers' messaging stream at four timepoints over a 2‐year period (2021–2023). This included the baseline at timepoint 1 (T1) collected upon recruitment, timepoint 2 (T2) collected at 6 months into the intervention, timepoint 3 (T3) at 1 year into the intervention, and timepoint 4 (T4) 2 years, or upon conclusion of the intervention.

Data from CHQ were available on the total number of participants in the overall intervention and the streams in which they were enrolled. CHQ program data also included information on text messaging frequency. However, no demographic information was collected for participants who did not opt to participate in the research.

### 2.2 Sampling

Research participants were recruited through convenience sampling over a 10‐month period (March–December 2021). Mothers could register for C2u only after giving birth, using an online portal hosted by CHQ HHS, and could opt into the research component during registration. Eligibility for C2u required participants to have had a full‐term baby. While mothers who gave birth before C2u launched in March 2021 were still eligible to register for the program, participation in the research required that their baby was 4 months old or younger at the time of enrolment.

The intervention was initially available to parents residing in 42 targeted suburbs within the Greater Brisbane area of CHQ HHS, identified as having high developmental vulnerabilities according to 2018 Australian Early Development Census data [37]. However, the decision was later made to extend the intervention statewide in Queensland, inviting all eligible participants to join both C2u and the research, thereby making the intervention universal. This shift promotes equity of access and supports the idea of empowering all individuals to enhance their health.

C2u was promoted to families across Queensland during both the antenatal and post‐birth periods. For antenatal care, promotional materials were displayed in child health clinics within the targeted CHQ HHS suburbs, and fridge magnets were provided for potential participants to take home. These materials were also distributed to community partners, including Mission Australia's Communities for Children in Inala/Ipswich and the Southern Moreton Bay Islands Community Impact Initiative, as well as to Child Health Nurses, Midwives, and General Practitioners for recruitment during child health drop‐in clinics and appointments.

Post‐birth recruitment involved Child Health Nurses and community partners recommending C2u to mothers during visits. CHQ HHS also sent SMS invitations to families after their initial appointments with a Child Health Nurse. Additionally, posts on community partners' Facebook pages and the official CHQ Facebook page directed participants to the web registration page. Facebook advertising was employed to promote sign‐up for the broader C2u intervention.

### 2.3 Instrument

The online self‐reported survey included both closed and open‐ended questions. The survey was disseminated to participants at four different timepoints (T1–T4).

#### 2.3.1 Demographics

Demographic variables included age, gender, relationship status, ethnicity, living arrangement, employment status, recruitment method to C2u, first child, educational level achieved, and current annual household income ($AUD). Demographics were collected at T1, with specific variables (age, relationship status, living arrangement, employment status, educational level, and income) updated at T3 and T4.

#### 2.3.2 Parenting Confidence

Parenting confidence was assessed using the 15‐item Karitane Parenting Confidence Scale (KPCS) developed in Australia by Črnčec et al. [38]. This tool measures self‐efficacy in parents of infants up until 12 months, and was collected at T1, T2 and T3 in this study. The KPCS utilises a 4‐point Likert response scale which can be scored as 0, 1, 2 or 3, with not applicable items being scored 2. Scores are then summed to give a total score between the range of 0 and 45. Higher scores indicate greater confidence, with the clinical cut‐off being 39 or less for significantly low levels of parenting confidence.

#### 2.3.3 Social Support

An 8‐item measure of individual experience of social support, with two subscale measures of emotional support and instrumental/tangible support, was collected using the Modified Medical Outcome Social Support survey (mMOS‐SS) [39, 40] at T1, T2, T3 and T4. The mMOS‐SS utilises a 5‐point Likert response scale, which can be scored as 1, 2, 3, 4 and 5. Scores are then summed to give a total score between the range of 8–40. Scores were transformed to a 0–100 scale using the provided formula 100 × (observed score−minimum possible score)/(maximum possible score−minimum possible score). A higher score indicates more support.

Between T2–T4, participants responded to 8 statements about their relationships with their children, partner (if applicable), and awareness of local parent groups using a 5‐point Likert scale (1 = strongly disagree, 5 = strongly agree). One open‐ended question was asked to explore participants' experiences of support from C2u, including reflection on partner relationships (if applicable). While these measures were collected at T2 and T3, this study reports T4 data to capture participants' reflections after completing the full 24‐month intervention, providing insight into overall experiences. However, Likert response data from T2 and T3 are provided in Tables S1 and S2.

#### 2.3.4 Support Preventative Health Efforts

Three statements assessed the support C2u provided for participants' well‐being, using the same 5‐point Likert scale. Feeding practices were assessed through one statement, and three additional statements addressed the promotion of services, including child health and vaccination appointments. Two open‐ended questions explored support provided by C2u, including how the intervention assisted with parenting and the most useful things learned from participating. Although collected at earlier timepoints (T2 and T3), this study reports the data collected at T4, with earlier Likert scale responses reported in Tables S1 and S2. Child immunisation status was assessed at T4 by asking participants whether their child had received 2‐, 4‐, 6‐, 12‐ and 18‐month vaccinations.

#### 2.3.5 Satisfaction

Participants answered eight statements about their satisfaction with the C2u intervention, also using a 5‐point Likert scale. Participants were asked one open‐ended question about areas for improvement. Although these measures were also collected between T2 and T4, only T4 responses are reported. Earlier Likert responses (T2 and T3) are available in Tables S1 and S2.

### 2.4 Data Analysis

#### 2.4.1 Quantitative

Descriptive statistics were performed, with variables expressed as frequencies (percentages) for demographics, vaccination and agreeance statements. For statements with the strongly disagree to strongly agree Likert response frame, responses were merged into one singular response for strongly disagree and disagree due to small response numbers (< 10). For consistency, agree and strongly agree responses were also merged into one response. In addition, parental confidence was descriptively analysed to identify the proportion of participants (*n*, %) scoring ≤ 39 on the KPCS across T1–T3. Age range at each time point was reported, alongside the mean (M) and standard deviation (SD). Analyses were performed using IBM SPSS version 29.0.2.

Two of the objectives of this study focused on whether C2u supported parental confidence and social support. To investigate this, data normality was first assessed for parental confidence (KPCS) and social support (mMOS‐SS) to ensure the use of appropriate statistical methods. Initial assessment was conducted using quantile–quantile (Q–Q) plots of the residuals with 95% confidence bands, which revealed deviations from normality. The Kolmogorov–Smirnov test was then applied to both variables, with results indicating *p* < 0.001, confirming that the data were not normally distributed.

Consequently, the trajectories of total parenting confidence/social support scores following C2u were modelled using linear quantile mixed effects models (LQMMs). Results for the 25th, 50th and 75th quantiles are reported, although the 50th percentile is the main interest. Time (months) was included as a fixed effect, as was baseline total parenting confidence/social support to account for regression to the mean. Time was also included as a random effect, to account for the lack of independence caused by repeated measurements on patients. Time zero was defined as baseline (upon recruitment). One model was used to estimate the average improvement in total confidence for the first 6 months (baseline defined as 0 months) and another to estimate the average improvement in total confidence for the second 6 months (baseline defined as 6 months), due to the change not being constant over these two time periods. For social support, only one model was used for the average improvement in social support score for the whole 2‐year follow‐up period since there was no evidence to suggest a violation of the linearity assumption. Standard error (SE), Confidence intervals (CI), interquartile range (IQR), and exact *p* values are reported, and the use of statistical significance terminology has been avoided [41, 42].

Additional descriptive models [43], each adding a single participant demographic characteristic and its interaction with time, were used to investigate whether total parenting confidence or social support scores, and/or their rate of improvement, differed according to maternal age (years), relationship status (married/partnered = 0, single/separated/widowed = 1), parity (first child: no = 0, yes = 1), educational achievement (did not complete high school/completed high school = 0, diploma/trade certificate = 1, university degree = 2), or household income (< 80 000 = 0, 80 001–120 000 = 1, > 120 000 = 2). The interaction indicating how the rate of improvement differed across participant subgroups was the main interest, thus, for brevity interaction coefficients and 95% CIs are reported in the manuscript, but the results of the full model with main effects and the intercept are available in Tables S3–S5. All LQMM analyses was conducted using R version 4.4.0 [44].

#### 2.4.2 Qualitative

Open‐ended survey responses from T2, T3 and T4 were analysed using qualitative content analysis [45]. This approach is appropriate for brief survey responses, allowing systematic identification and organisation of patterns while preserving participant meaning. Responses were read repeatedly to familiarise the research team with the data and to identify content units. An inductive, iterative process was used to generate codes, which were then manually clustered into themes and subthemes. Final themes were determined through discussion among authors (L.I.J., A.W. and L.V.). Six overarching themes were identified: ‘Reassurance on development’, ‘Supportive Network’, ‘Mothers' health’, ‘Feeding practices’, ‘Engagement with services’ and ‘Satisfaction’.

Participant demographics (age range, first‐time mother vs. multiparous and partner status) are reported alongside illustrative quotes, with each quote labelled by survey timepoint (T2, T3 or T4) to provide temporal context. While the number of participants responding to each item is reported, the analysis focused on identifying patterns and themes across timepoints rather than quantifying the frequency of comments supporting each theme.

### 2.5 Ethical Considerations

Ethics approval for the study was granted by the relevant Human Research Ethics Committees (CHQ HHS [LNR/20/QCHQ/71980] and Griffith University [2021/015]). Prior to participation, all participants were presented with an online parent/guardian information sheet embedded within the CHQ‐hosted registration page. This sheet outlined the purpose of the research, expected benefits and minimal risks, data use and confidentiality, and the voluntary nature of participation, including the right to withdraw at any time. Participants indicated their informed consent by electronically selecting a checkbox, which was required before proceeding with the research component.

## 3 Results

A total of 7946 participants were recruited for the 2021–2023 iteration of C2u, with 89.5% enrolled in the mothers' messaging stream, 9% in the fathers/carers stream, and 1.5% in Aboriginal and/or Torres Strait Islander mothers or fathers/carers streams. At T1 (recruitment), 1007 mothers signed up to participate in the C2u research study. Table 1 shows that most participants joined C2u via Facebook (59%), followed by text messages from CHQ HHS (10.3%). At T2 (6 months), 577 mothers completed the follow‐up survey, representing a retention rate of 57% from baseline. At T3 (12 months), 346 mothers who had completed both T1 and T2 participated, corresponding to a 60% retention rate from T2. At T4 (24 months), 151 mothers who had completed T1, T2, and T3 participated, reflecting a retention rate of 44% from T3.

**TABLE 1:** Recruitment method of C2u research participants at T1 (*n* = 1005, 2 missing).

Recruitment method	*N*	%
Facebook	593	59
Text messages from CHQ	104	10.3
Child Health Drop‐in Clinic	82	8.2
Friends and family	53	5.3
Child Health Appointment	49	4.9
Antenatal Care	48	4.8
Social media (other than Facebook)	47	4.7
Community partners	17	1.7
Other (internet search, through employer)	12	1.2

Responses to the open‐ended questions varied by item and timepoint. At T2, 262 (45.4%) mothers responded to the question about the most useful things they learned, 327 (56.7%) responded regarding areas for improvement, 282 (48.9%) responded regarding how C2u assisted with parenting, and 325 of the 533 who reported having a partner (61%) responded regarding support for their relationship with their partner. At T3, 208 mothers (60.1%) responded about useful learnings, 231 (66.8%) about areas for improvement, 218 (63%) about parenting support, and 212 of the 316 partnered mothers (67%) about relationship support. At T4, 69 mothers (45.7%) responded about useful learnings, 45 (29.8%) about areas for improvement, 59 (39.1%) about parenting support, and 48 of the 142 partnered mothers (33.8%) about relationship support. Responses such as ‘not applicable’ or ‘no suggestions’ were excluded from these counts.

### 3.1 Participant Characteristics

Demographic characteristics are shown in Table 2. The mean age of participants at recruitment was 32.22 (SD ± 4.25). The majority of participants were married or in a partnership and identified as Caucasian. Approximately two‐thirds of participants enrolled in C2u with their first child. Educational levels varied, with the majority of participants having a diploma, trade certificate, or university degree. The combined annual household income also varied, although the majority of participants reported household earnings of more than $120 001 at T4.

**TABLE 2:** Demographic characteristics of participants.

Demographics	T1 (%) (*n* = 1007)		T2 (%) (*n* = 577)		T3 (%) (*n* = 346)		T4 (%) (*n* = 151)	
	*n*	%	*n*	%	*n*	%	*n*	%
Age (range 18–43)	M = 32.22 (SD ± 4.25)		M = 32.28 (SD ± 4.21)		M = 33.28 (SD ± 4.20)		M = 34.31 (SD ± 4.3)	
Gender								
Female	996	98.9	573	99.3	344	99.4	150	99.3
Another gender*	11	1.1	4	0.7	2	0.6	1	0.7
Relationship status								
Married or partnership	966	95.9	553	95.8	326	94.2	142	94.0
Single/separated/widow	41	4.1	24	4.2	20	5.8	9	4.2
Ethnicity								
Caucasian	867	86.1	515	89.3	315	91.0	139	92.1
Asian	85	8.4	42	7.3	21	6.1	8	5.3
Aboriginal and/or Torres Strait Islander	26	2.6	10	1.7	4	1.2	1	0.7
African, Hispanic, Mixed race, Pacific Islander^a^, PNTS^b^	22	2.2	10	1.7	6	1.8	3	2.1
Living arrangement								
With partner	954	94.7	544	94.3	326	94.2	139	92.1
Alone	32	3.2	19	3.3	14	4.0	8	5.3
With parents, shared house or parents and partner	21	2.1	14	2.4	6	1.7	4	2.6
Employment status								
Maternity leave	723	71.8	427	74.0	81	23.4	19	12.6
Full time	131	13.0	70	12.1	78	22.5	46	30.5
Part time	41	4.1	20	3.5	135	39.0	62	41.1
Casual	18	1.8	10	1.7	15	4.3	12	7.9
Unemployed	75	7.4	36	6.2	29	8.4	10	6.6
Students or carer	19	1.9	14	2.4	8	2.3	2	1.3
First child								
Yes	662	65.7	388	67.2	242	69.9	107	70.9
No	345	34.3	189	32.8	104	30.1	44	29.1
Educational level								
Did not complete high school or high school certificate	126	12.5	62	10.7	36	10.4	13	8.6
Diploma or trade	189	18.8	100	17.3	55	15.8	27	17.9
University degree (including postgraduate)	692	68.7	415	72	255	73.7	111	73.5
Annual household income ($AUD)								
< 80 000	149	14.8	74	12.9	58	16.8	9	6.0
80 001–120 000	279	27.7	168	29.1	110	31.8	39	25.8
> 120 001	533	52.9	308	53.4	178	51.4	96	63.6
PNTS^b^	46	4.6	27	4.7	—	—	7	4.6

^a^ Due to small response numbers, African, Hispanic, Mixed Race, Pacific Islander and PNTS have been combined.

^b^ PNTS – Prefer not to say.

* Due to small response numbers, “Another gender” includes responses from participants who identified as male, did not identify with a particular gender, or preferred not to say.

### 3.2 Parenting Confidence

The median total confidence score was 38.0 at T1 (IQR: 33.0–42.0), which increased to 41.0 (IQR: 38.0–43.0) at T3 (see Table 3). At T1, 63.9% of participants scored ≤ 39 (indicating low parenting confidence), compared with 36.7% at T2 and 34.1% at T3. When restricting T1/T2 analyses to participants who completed all three timepoints, percentages were similar at T1 (64.7%) and T2 (37.0%). The median total parenting confidence increased by an average of 0.376 points per month during the first 6 months (95% CI 0.320–0.432, *p* < 0.0001), and by 0.085 points per month during the second 6 months (95% CI 0.029–0.141, *p* = 0.003). These findings indicate that total parenting confidence improved over the study period, with a larger increase observed during the first 6 months compared to the second.

**TABLE 3:** Descriptive statistics and linear quantile mixed model results for parenting confidence and social support outcomes.

Descriptive statistics					Linear quantile mixed model rate of change estimates per month									
Outcome	Timepoint	*N*	% ≤ 39^a^	Median (IQR)	Time period (months)	25th Quartile Estimate (95% CI)	SE	*p*	50th Quartile^b^ Estimate (95% CI)	SE	*p*	75th Quartile Estimate (95% CI)	SE	*p*
Parent confidence	1	1007	63.9%	38.0 (33.0–42.0)	0–6	0.374 (0.319, 0.429)	0.027	< 0.0001	0.376 (0.320, 0.432)	0.028	< 0.0001	0.377 (0.320, 0.435)	0.029	< 0.0001
	2	577	36.7%	41.0 (38.0–43.0)										
	3	346	34.1%	41.0 (38.0–43.0)	6–12	0.078 (0.012, 0.145)	0.033	0.023	0.085 (0.029, 0.141)	0.028	0.003	0.089 (0.034, 0.144)	0.027	0.002
Social support	1	1007	—	78.1 (65.6–93.8)	0–24	−0.121 (−0.281, 0.038)	0.079	0.132	0.011 (−0.096, 0.118)	0.053	0.837	0.013 (−0.099, 0.125)	0.056	0.813
	2	577	—	75.0 (59.4–90.6)										
	3	346	—	75.0 (65.6–93.8)										
	4	151	—	75.0 (62.5–90.6)										

^a^ Scores on the Karitane Parenting Confidence Scale range from 0 to 45, with higher scores indicating greater parenting confidence. A score of 39 or below is considered to indicate low parenting confidence and falls below the established clinical threshold.

^b^ Q50 (median) estimates are the primary focus of interpretation.

Although participants were not directly asked about their perceived levels of parenting confidence, feedback indicated that the messages from C2u provided reassurance about their child's development. This reassurance provided participants with confidence that, ‘what was happening with my child was normal’ (see Table 4 for further qualitative insights). However, some participants shared that messages need to account for if their child was not meeting normal development milestones. Nonetheless, participants shared that they felt the messages came through at a time when they were experiencing the subject, or just about to, which would help them realise they were ‘on track’.

I often would find myself thinking about changes in my child's behaviour and reflecting if it was due to her development. Then within a few days of the thought and new behaviour I'd receive a C2u message that discussed exactly what I'd been noticing. I found it reassuring and validating that what I was observing was likely normal development. I found it affirming that I was doing a good job if my child was demonstrating normal behaviours for her age and that I was picking up on the changes (*T4, 30 –34 years, first time mother, partnered*).

This reassurance seemed to be particularly useful for first‐time mothers, or mothers who had longer time periods between their own children (i.e., 5 years between children), with one first‐time mum sharing that they felt the messages comforting that they were getting better as a mother and made them ‘feel seen’. This finding is supported by Table 5 (see Tables S3–S5 for full LQMM results), which shows that first‐time parents experienced faster increases in median parenting confidence during the first 6 months compared with parents who already had children (0.250 additional points per month increase, 95% CI: 0.109, 0.391, *p* = 0.001). First‐time parents continued to show faster improvements in median confidence over months 6–12 (0.162 additional points per month increase, 95% CI: 0.050, 0.275, *p* = 0.006). Other demographic characteristics showed smaller or less consistent relationships with the rate of change in confidence. However, for months 6–12, participants with university‐level education showed modestly faster improvements in median parenting confidence compared to those who did not finish high school/high school certificate education level (0.149 points per month, 95% CI: 0.009, 0.288, *p* = 0.038).

**TABLE 4:** Qualitative insights across T2–T4.

Theme	Sub‐theme	Associated quotes
Reassurance on development	Timely messages on developmental stages	Given me confidence and support in what was happening with my child was normal, emotionally and developmentally and giving me useful ideas in interacting with my child (T4, 35 years+, first‐time mother, partnered) I often would find myself thinking about changes in my child's behaviour and reflecting if it was due to her development. Then within a few days of the thought and new behaviour I'd receive a C2U message that discussed exactly what I'd been noticing. I found it reassuring and validating that what I was observing was likely normal development. I found it affirming that I was doing a good job if my child was demonstrating normal behaviours for her age and that I was picking up on the changes (T4, 30–34 years, first‐time mother, partnered) It has helped me to realise I am on track. I've found the messages come through at a time we are experiencing the subject or just about to (T4, 35 years+, first‐time mother, partnered) Honestly, they just seemed to come when I was feeling a little lost or overwhelmed. They really encouraged me and while no one thing stands out they have taught or re‐affirmed a lot for me (T3, 25–29 years, first‐time mother, partnered)
	Guidance for new and returning parents	As a first‐time mum, it reassured me that her development was on track, that I was getting better at being a mum and it made me feel seen (T2, 25–29, first‐time mother, partnered) Even as a second time parent because I had 5 years between kids some of them are a good reminder about what to do when with your child to encourage certain areas of development (T4, 35 years+, multiparous, partnered)
	Need to account for differences	More reassurance if child isn't hitting the milestone as expected. It can be a very broad range for milestones, and most messages are ‘get it checked out’ rather than reassurance that all children's development is different (T4, 30–34 years, first‐time mother, partnered) There needs to be an opt out if your child is not meeting normal development. These texts became incredibly upsetting and unhelpful reminders that my child was not developing normally (T4, 35 years+, first‐time mother, partnered) Be careful with milestones, if a child isn't quite there yet, it can cause some panic, something like ‘between now and next month, you'll likely see XXXX’ rather than ‘by now you'll be doing XXX’ (T4, 35 years+, first‐time mother, partnered)
Supportive network	Stimulating discussion between partners	I'm not sure it's had a huge impact on our relationship. But he's also found the knowledge I've shared from the messages helpful (T4, 30–34 years, first‐time mother, partnered) I felt it was most valuable in the very early days of becoming a mother for the first time. It was great to share the texts with my husband so we both felt more educated (T4, 30–34 years, first‐time mother, partnered) I loved getting positive texts with ideas and words of encouragement. It stimulated conversations with my husband and extended family. It was just a really positive message to get among all the noise of social media and back to work, and so forth, it was a very positive experience for our family as first‐time parents (T4, 35 years+, first‐time mother, partnered) Kept us aligned and laughing at the little things. He would often say ‘*baby* texted me today and says she needs help brushing her teeth’ or similar things. Good reminder that all couples are navigating the same challenges (T4, 35 years+, first‐time mother, partnered) We really enjoyed sharing the text messages we received. It's great that they're tailored to mum and dad. They've been starting points for conversations about our daughter and helped us come up with an aligned approach to parenting (T3, 30–34 years, first‐time mother, partnered)

	Need for connection with partner	Keep working on supporting relationships with partners and other children particularly for a second child, as this is a very different experience (T4, 35 years+, multiparous, partnered) This is an area that could be improved. I have never felt more disconnected than I do now. When there is more than one child in the house you can lose sight of each other (T4, 35 years+, multiparous, partnered) My husband signed up to the connecting2u project and I believe it has helped us both in positive ways. For example, it reminded us to be a team and help each other out. It reminded us that caring for each other is just as important than caring for baby (T2, 25–29, first‐time mother, partnered) It's reminded me to focus on my relationship with my husband and to not be hard on myself as a mother (T2, 35 years+, first‐time mother, partnered)
	Extra layer of support	All those messages popping in my inbox helped me a lot about what to expect, what to do next. They were so supportive that it brings a smile on my face with all reminders (T4, 25–29, multiparous, partnered) It was good at the start, made me feel like I had an extra layer of support. It was good reminder for child checks and immunisations (T4, 35 years+, first‐time mother, partnered) That I'm not alone and that we are on track! (T4, 35 years+, first‐time mother, partnered) It was just nice to get a little reminder of what I should be doing, and it really did help me feel a little more supported at times (T4, 30–34 years, first‐time mother, partnered) It has reminded me about how to bond with my child (T3, 30–34 years, multiparous, partnered) It's weird how emotional it can be reading an affirming C2u text message, considering it's a generic message. But they are so validating! Sometimes I tear up. And it's spooky how accurate they are. For example, the message about returning to work came in the week that I returned to work (T3, 30–34 years, first‐time mother, partnered) The text messages were really great for reminding me that I am connected and that there is support around me if I needed it. It was nice to receive encouragement, and the text messages made me smile when I received them (T2, 30–34 years, multiparous, single)
Awareness of ‘mums’ groups	I loved receiving the texts every week. They gave me great play ideas…helped me find a mums group and made me feel like a good mum (T4, 30–34 years, first‐time mother, partnered) Would also be good to be able to learn from others in the network so maybe an add on could be a messenger chat or private Facebook group for parents with same aged kids (T4, 35 years+, multiparous, partnered) Possibly making any suggestions to social events, play groups to the location you are in potentially (T4, 25–29, first‐time mother, partnered) More links to localised play groups and child health services (T3, 30–34 years, first‐time mother, partnered) Provide a way of connecting to other mothers with similar aged children in the local area. There is little sense of community these days which makes it hard as a mother at home with 2 young children. It is very isolating (T3, 25–29, multiparous, partnered) Some option to use it as an initial way to meet other parents with babies around the same age (would be useful). Maybe you could opt in to share your location and to chat with other parents within a certain radius. Bit like how online dating apps work but for families to help build your village! (T2, 40, first‐time mother, partnered) Link with other mothers in the local area (would be helpful). I could not attend a mother's group because of Covid and this is my second baby—I couldn't manage to take both to a mother's group even if one was available. Would be good to set up virtual mothers' groups because it is very isolating being at home with 2 children, especially during covid when face to face groups are being cancelled (T2, 30–34 years, multiparous, partnered)
Mothers' health	Self‐care	The text messages were great reminders to look after myself and always made me smile when I received them as they reminded me that there are always people around for support when I need it. It helped me with my confidence also (T3, 30–34 years, first‐time mother, partnered) I liked the prompts of the messages for self‐care. Even just encouraging parents to observe their children, journal or generally be more mindful and enjoy the parenting journey was a positive influence in my first 2 years as a mother (T4, 35 years+, first‐time mother, partnered) Perhaps me, but I found the self‐care recommendations a bit shallow. The vibe I remember was ‘just take care of yourself’ which is obviously easier said than done. More concrete suggestions and acknowledgement/validating of emotions would be helpful (T4, 30–34 years, first‐time mother, partnered)
Feeding practices		Starting solids was so stressful for me. I was so anxious. But getting the text message about food suggestions and where to get more info made the process so much easier! (T4, 25–29, first‐time mother, partnered) In the early days information on introducing food and then things like children expressing their feelings/tantrums were helpful (T4, 30–34 years, first‐time mother, partnered)
Engagement with services	Health services	Good for reminding me about health checks and developmental stages! The messages have helped me feel supported in a way where it's a gentle reminder every now and then to help my parenting skills. For example, giving ideas to try lentils for food, games with baby, and so forth (T2, 35+, multiparous, partnered) Reminded me to see things from babies' perspective and how he would be feeling, reminded me to book in for health checks and vaccinations (T4, 35+, multiparous, separated)
	Immunisations	It reminded me to book in immunisations and health checks and the links were always useful for the stage/age we were in (T4, 30–34 years, first‐time mother, partnered) Reminder re important things, for example, immunisations (T2, 25–29, multiparous, partnered)
Satisfaction	Overall satisfaction	Honestly all of it was useful, I was so excited to open the texts every time they came in to see what I would learn next (T4, 30–34 years, first‐time mother, partnered) I liked the tone. I felt it helped to give my baby a voice before he could speak (T4, 30–34 years, first‐time mother, partnered) Wasn't so much new things learnt but reminders of things I already knew from my first (T4, 35+, multiparous, partnered) More than anything it has been like a friend, gently reminding me to book appointments, prioritise self‐care, reiterate feeding practices and remind me of developmental milestones. I had read a lot while pregnant so knew about most of what the texts said anyway, but I think it is a great service, especially for those who don't prepare much or who are quite young and maybe overwhelmed by parenting (T3, 30–34 years, first‐time mother, partnered)
	Increase frequency	I really enjoyed and appreciated the messages. I would have been happy to receive more. Although, I do appreciate that if they were too frequent people may pay less attention to them. I certainly used the links provided (T4, 30–34 years, first‐time mother, partnered) I would suggest if the number of texts will increase. It would be great help. And something related to siblings. How to bond with older siblings of new baby? It would've helped (T4, 25–29, multiparous, partnered)

**TABLE 5:** LQMM covariate‐time interaction results for rate of change in parenting confidence and social support.^a^

Covariate	Q25 Estimate (95% CI)	SE	*p*	Q50^b^ Estimate (95% CI)	SE	*p*	Q75 Estimate (95% CI)	SE	*p*
Parenting Confidence (0–6 months)									
Maternal age	0.003 (−0.011, 0.017)	0.007	0.642	0.007 (−0.008, 0.022)	0.007	0.323	0.011 (−0.004, 0.026)	0.007	0.163
Relationship	0.081 (−0.239, 0.401)	0.159	0.613	0.082 (−0.239, 0.402)	0.159	0.611	0.082 (−0.237, 0.401)	0.159	0.606
First child	0.240 (0.082, 0.399)	0.079	0.004	0.250 (0.109, 0.391)	0.070	0.001	0.261 (0.115, 0.407)	0.073	0.001
Education									
Diploma/Trade certificate	0.061 (−0.229, 0.350)	0.144	0.675	0.060 (−0.232, 0.353)	0.146	0.680	0.062 (−0.227, 0.350)	0.144	0.669
University	0.118 (−0.173, 0.410)	0.145	0.419	0.119 (−0.178, 0.417)	0.148	0.424	0.140 (−0.155, 0.435)	0.147	0.346
Income									
80–120 K	0.131 (−0.057, 0.320)	0.094	0.168	0.155 (−0.051, 0.361)	0.103	0.138	0.154 (−0.054, 0.362)	0.104	0.143
> 120 K	0.163 (−0.048, 0.375)	0.105	0.127	0.170 (−0.035, 0.376)	0.102	0.101	0.172 (−0.040, 0.384)	0.105	0.109
Parenting Confidence (6–12 months)									
Maternal age	−0.004 (−0.017, 0.010)	0.007	0.605	−0.001 (−0.015, 0.012)	0.007	0.846	0.000 (−0.013, 0.013)	0.007	0.993
Relationship	−0.109 (−0.369, 0.150)	0.129	0.401	−0.110 (−0.369, 0.149)	0.129	0.399	−0.109 (−0.368, 0.150)	0.129	0.402
First child	0.147 (0.033, 0.260)	0.056	0.012	0.162 (0.050, 0.275)	0.056	0.006	0.165 (0.051, 0.278)	0.056	0.005
Education									
Diploma/Trade certificate	0.070 (−0.097, 0.238)	0.083	0.403	0.076 (−0.093, 0.245)	0.084	0.372	0.075 (−0.093, 0.243)	0.084	0.373
University	0.124 (−0.016, 0.265)	0.070	0.081	0.149 (0.009, 0.288)	0.070	0.038	0.155 (0.016, 0.295)	0.069	0.030
Income									
80–120 K	0.044 (−0.100, 0.188)	0.072	0.539	0.048 (−0.096, 0.192)	0.072	0.506	0.066 (−0.077, 0.209)	0.071	0.359
> 120 K	0.064 (−0.078, 0.206)	0.070	0.370	0.079 (−0.062, 0.221)	0.070	0.264	0.076 (−0.066, 0.217)	0.070	0.288
Social Support (0–24 months)									
Maternal age	−0.012 (−0.032, 0.008)	0.01	0.228	−0.008 (−0.021, 0.006)	0.007	0.274	−0.007 (−0.029, 0.014)	0.011	0.51
Relationship	0.013 (−0.382, 0.408)	0.197	0.947	−0.022 (−0.428, 0.384)	0.202	0.913	−0.022 (−0.441, 0.397)	0.209	0.915
First child	−0.280 (−0.578, 0.018)	0.148	0.065	−0.139 (−0.410, 0.132)	0.135	0.308	−0.112 (−0.367, 0.144)	0.127	0.384
Education									
Diploma/Trade certificate	−0.295 (−0.607, 0.017)	0.155	0.063	−0.282 (−0.558, −0.007)	0.137	0.045	−0.215 (−0.489, 0.060)	0.137	0.123
University	−0.258 (−0.577, 0.061)	0.159	0.110	−0.228 (−0.467, 0.012)	0.119	0.062	−0.245 (−0.501, 0.011)	0.127	0.061
Income									
80–120 K	0.089 (−0.160, 0.338)	0.124	0.476	0.212 (−0.020, 0.444)	0.115	0.072	0.240 (−0.002, 0.482)	0.120	0.052
> 120 K	0.148 (−0.126, 0.422)	0.136	0.283	0.135 (−0.113, 0.384)	0.124	0.280	0.159 (−0.095, 0.412)	0.126	0.214

^a^ Interaction with time: All coefficients presented in the table represent the interaction between the covariate and time in months (Covariate × Timepoint in Months). These coefficients indicate whether the rate of change in the outcome (parenting confidence 0–6 months, parenting confidence 6–12 months and social support 0–24 months) differs according to the covariate. To aid interpretation, full model results, including intercepts, main effects, baseline scores, and interactions, are provided in the Tables S3–S6, allowing readers to contextualise the rate of change relative to participants' starting points.

^b^ Q50 (median) estimates are the primary focus of interpretation.

### 3.3 Social Support

Although some participants reported feeling ‘seen’, the median total social support score was 78.1 at T1 (IQR: 65.6–93.8) and decreased slightly to 75.0 at T4 (IQR: 62.5–90.6) (see Table 3). Results from the LQMM showed little evidence of an improvement in median total social support, with an estimated average improvement of 0.011% per month (95% CI: −0.096–0.118, *p* = 0.837) during the 2‐year follow‐up period. The apparent discrepancy between the slight decrease in median scores from T1 to T4 and the near‐zero positive change estimated by the model is likely largely driven by the fact that the regression model includes random effects to account for the lack of independence between participant repeated measurements, with the aim of estimating average ‘within‐person’ changes. For this reason, the results of the regression model in Table 3 should be prioritised, and the median and IQR results should be considered descriptive estimates of median scores at different timepoints.

Qualitative responses revealed varied perceptions of C2u supporting relationships with their partner. While some participants noted that C2u prompted meaningful conversations with their partners, others reported that they believed it did not influence their relationship. This variation is reflected in the quantitative findings: as shown in Table 5, the rate of change in social support did not meaningfully differ by relationship status (−0.022, 95% CI: −0.428, 0.384, *p* = 0.913). Nevertheless, several participants noted that the messages reminded them to engage with their partners, and one participant, whose partner received the partner stream of C2u messages, shared:

Kept us aligned and laughing at the little things. He would often say ‘baby texted me today and says she needs help brushing her teeth’ or similar things. Good reminder that all couples are navigating the same challenges (*T4, 35 years+, first time mother, partnered*).

This diversity in experiences was also reflected in the agreeance statements at T4 (see Table 6). While only 16.9% disagreed that the text messages helped their relationship with their partner or support person, nearly half (48.4%) neither agreed nor disagreed. Qualitative responses suggested that for parents of multiple children, further refinement of C2u messaging may be needed to better support partner relationships, with one participant sharing that, ‘when there is more than one child in the house you can lose sight of each other’ (*T4, 35 years+, multiparous, partnered*).

**TABLE 6:** Statements of support and satisfaction of C2u at T4 (*n* = 151).

Statement	Disagree		Neither		Agree	
	*N*	%	*N*	%	*N*	%
Support of relationships						
The text messages helped my relationship with my partner/support person (*n* = 124)	21	16.9	60	48.3	43	34.7
2 Helped me feel emotionally supported when returning to daily activities (e.g., returning to work)	13	8.6	46	30.5	92	60.9
3 I have shared the C2u information with others (e.g., partner, family, friends)	25	16.6	24	15.9	102	67.6
4 Helped me feel closer to my child	9	6.0	39	25.8	103	68.2
5 Increased my awareness of available social support groups in my area (e.g., library playgroups)	8	5.3	37	24.5	106	70.2
6 Reminded me to take part in social support groups in my area such as mothers/parents' groups and library playgroups	3	2.0	29	19.2	119	78.9
7 Gave me ideas for interacting with my child (such as playing and reading with my child)	2	1.3	22	14.6	127	84.2
8 The text messages were good for my relationship with my child	2	1.3	22	14.6	127	84.2
Mothers' wellbeing						
Encouraged me to make healthier choices	13	8.6	42	27.8	96	63.5
2 Made me feel more prepared for parenting than what I thought I would be without the text messages	11	7.3	42	27.8	98	64.9
3 Reminded me to look after myself during these first 2 years of parenting	1	0.7	11	7.3	139	92.1
Feeding practices						
Gave me information about feeding practices that I have acted upon for my child	11	7.3	29	19.2	111	73.5
Promotion of services						
Made it easy to book in for the child health appointments	11	7.3	60	39.7	80	53.0
2 The text messages reminded me to book my child for vaccinations	5	3.3	19	12.6	127	84.1
3 Reminded me when my child's health check appointment was due	3	2.0	10	6.6	138	91.4
Satisfaction with C2u						
The text messages helped me to feel supported	4	2.6	12	7.9	135	89.4
2 I found the C2u text messages helpful	1	0.7	12	7.9	138	91.4
3 I used the web links in the text messages to find more information	11	7.3	28	18.5	112	64.2
4 I enjoyed being a part of C2u	1	0.7	8	5.3	142	94.0
5 I would recommend C2u to other parents	—	—	7	4.6	144	95.4
6 I understood all of the text messages	—	—	4	2.6	147	97.4
7 I liked how many text messages I receive	1	0.7	6	4.0	144	95.4
8 I was happy with the topics of the text messages	1	0.7	2	1.3	148	98.1

Other covariates, including maternal age (0.008, 95% CI: −0.021, 0.006, *p* = 0.274), first child status (−0.139, 95% CI: −0.410, 0.132, *p* = 0.308), household income (80–120 K: 0.212, 95% CI: −0.020, 0.444, *p* = 0.072, > 120 K: 0.135, 95% CI: −0.113, 0.384, *p* = 0.280), and education (University: –0.228, 95% CI: −0.467, 0.012, *p* = 0.062), also showed no meaningful differences in the rate of change over time. Mothers with a diploma or trade certificate showed a slightly slower rate of change in median social support compared with those who had completed high school or less (−0.282, 95% CI: −0.558, −0.007, *p* = 0.045), although the magnitude of the difference is small.

Separate from these findings, many participants described C2u as an ‘extra layer of support’. For example, one participant reflected, ‘all those messages popping in my inbox helped me a lot about what to expect next (*T4, 25–29, multiparous, partnered*). Some participants also shared that it reminded them about how to bond with their child. These sentiments were mirrored in the agreeance statements, with 91.4% agreeing that C2u gave them ideas for interacting with their child, and 84.2% agreeing that the text messages were good for the relationship with their child. Some participants also expressed interest in guidance on supporting sibling relationships, particularly in managing interactions between older children and a new baby.

Finally, C2u appeared to increase awareness of local support networks such as mother's groups. For example, 70.2% agreed that C2u increased their awareness of available social support groups in their area, and 79.9% agreed that it reminded them to participate in such groups. Many participants expressed interest in receiving more content that connected them to their local community. Suggestions included messages linking to nearby events or using platforms like Facebook to create groups where mothers with similarly aged children could connect and share experiences.

### 3.4 Support Preventative Health

#### 3.4.1 Mothers' Overall Well‐Being

There were positive results regarding C2u supporting mothers' overall well‐being. The findings suggest that this centred around self‐care. Within the T4 agreeance statements, 91.2% of participants agreed that C2u reminded them to look after themselves during the first 2 years of parenting. These findings were mirrored within qualitative findings, with one participant, who had multiple children, pointing out that for them it was in fact the reminders for self‐care that were the most useful messages to receive. In contrast, a first‐time mother noted that they would have liked ‘more concrete suggestions’, acknowledging that sometimes self‐care was ‘easier said than done’. Nonetheless, there was an overall positive response, with several participants sharing that the messages acted as a reminder to ‘take time out’.

I liked the prompts of the messages for self‐care. Even just encouraging parents to observe their children, journal or generally be more mindful and enjoy the parenting journey was a positive influence in my first two years as a mother (*T4, 35 years+, first‐time mother, partnered*).

#### 3.4.2 Encouraging Feeding Practices

Feeding practices emerged as a key theme from the qualitative findings. Participants shared that this was an area that they had found supportive, particularly in the early days regarding information on introducing food. For example, one participant shared that, ‘starting solids was so stressful for me. I was so anxious. But getting the text message about food suggestions and where to get more info made the process so much easier!’ (*T4, 25–29, first‐time mother, partnered*). This was mirrored within the T4 agreeance statement with 73.5% agreeing that C2u gave them information about feeding practice that they had acted upon.

#### 3.4.3 Promoting Engagement With Health Services

The promotion of engagement with health services had varying success. For example, 91.4% of participants at T4 indicated that the messages had reminded them when their child's health check appointment was due, although only 53% agreed that it made it easier for them to book in for the child health appointments. Certainly, the messages acting as a reminder were mirrored within the qualitative findings, reminding participants about booking in health checks and vaccinations. Of note, 100% of participants at T4 indicated that their child had received their 2, 4‐, 6‐, 12‐ and 18‐month vaccinations, with 84.1% agreeing that the text messages had reminded them to book their child for vaccinations.

### 3.5 Satisfaction

Overall, participants at T4 reported high levels of satisfaction with C2u across all eight statements. For seven of the statements, agreement scores ranged from 89.4% to 98.1%, while a smaller proportion (64.2%) indicated that they used the web links in the text messages to access additional information. Notably, 98.1% of participants were satisfied with the topics of the messages, with several qualitative comments highlighting appreciation for the ‘voice of the baby’ messaging. One participant shared, ‘I liked the tone. I felt it helped to give my baby a voice before he could speak’ (*30–34 years, first‐time mother, partnered*). Satisfaction with the intervention is further supported by CHQ program opt‐out data. Over the 2‐year period, only 410 of the 7946 C2u participants (5.2%) chose to opt out, suggesting strong acceptability.

In terms of message frequency, 95.4% of participants indicated satisfaction with the number of text messages received. CHQ program data show that messaging frequency varied throughout the intervention: two to three messages per week during the first 3 months, one message per week for the following 9 months, and one message per fortnight for the remaining 12 months. Qualitative feedback suggests an appetite for increased message frequency, with several participants expressing a desire for more messages and an extension beyond the 2 years. Some participants also shared feelings of sadness when the intervention ended.

## 4 Discussion

Our findings suggest positive results following the 2021–2023 iteration of C2u. There was an increase in parenting confidence within the first 12 months, with a greater improvement seen within the first 6 months. Participants reported that C2u provided reassurance on their child's development. Although there was little evidence to suggest change in social support over the 2 years, participants shared that C2u encouraged conversations with their partner, improved their interaction with their child, and increased their awareness of local support groups, although there was a desire for more messages addressing these areas. Overall findings indicate that C2u supported preventative health efforts, with positive anecdotes across supporting mothers' well‐being, feeding practices, and the promotion of engagement with health services. Of note, 100% of participants at T4 indicated that their child had received their 2‐, 4‐, 6‐, 12‐ and 18‐month vaccinations, exceeding the corresponding age‐specific immunisation coverage in Queensland for 1‐ (2021: 94.08%, 2022: 93.11%), and 2‐year‐olds (2022: 91.60%, 2023: 90.77%) [46].

During the C2u intervention, median parental confidence, as measured by the KPCS, increased from 38.0 at baseline to 41.0 by 6 months, representing a meaningful shift from below to above the threshold for clinically low confidence. Our analyses revealed that first‐time parents experienced faster gains in confidence throughout the first 12 months of parenting, suggesting C2u may be particularly effective for parents navigating the transition to parenthood for the first time. This is consistent with evidence highlighting that first‐time parents often face unique challenges in adjusting to their new role [47]. Targeted supports during this period, such as C2u, may therefore act as an additional layer of reassurance and guidance, helping to strengthen parental confidence at a critical stage of early parenting.

Other interventions have also successfully improved parental confidence, both with in‐person [48] and text‐based approaches [49]. For example, in a 24‐week text‐based intervention, mothers received educational modules on their infant's cognitive, social, and emotional development, along with age‐appropriate play activities, and parental confidence, as measured by the KPCS, increased from 40.0 at 6 weeks to 41.9 at 24 weeks [49]. Our analyses suggested that participants with university‐level education experienced modestly faster improvements in confidence during months 6–12. While some research indicates that parents with higher levels of education tend to have greater knowledge of child development and effective parenting strategies [50, 51], further investigation is needed to understand how these factors may influence engagement with parenting interventions, for example, whether higher‐educated parents are more likely to apply the information provided, or whether certain delivery formats (e.g., SMS with links to videos or audio) are more effective for different socioeconomic groups. Importantly, other demographic factors were not strongly associated with changes in confidence, suggesting that C2u's benefits were similar across the participant group.

However, research by Pärtty et al. [52] also indicates that unsettled infant behaviour is linked to lower feelings of competence and self‐efficacy. Infants are more likely to exhibit unsettled behaviour between 0 and 6 months of age. Therefore, studies have found that KPCS scores typically increase over time. For example, Khajehei and Finch [53] found that KPCS scores averaged 35.2 for parents of infants aged 0–6 months and rose to 39.4 for those with infants aged 7–12 months. Similarly, Kristensen et al. [54] observed a significant increase in KPCS scores among first‐time mothers, from 40.98 at 2 months postpartum to 41.78 at 6 months postpartum. Therefore, while we noted in our study an increase in parental confidence, this improvement may be influenced by other factors, such as the child's growing age. As C2u continues in Queensland, it would be valuable to assess its impact on parental confidence through a randomised controlled trial (RCT), or an observational study emulating a target trial, to better understand its effectiveness.

Median social support scores, measured using the mMOS‐SS, showed little change from the start of the intervention (78.1) to 2 years later (75.0), with the LQMM indicating a negligible increase in the median (0.011 points per month; 95% CI: −0.10 to 0.12, *p* = 0.836) over the follow‐up period. Although previous research has shown that interventions during the postpartum period can enhance social support, for example, through internet‐based programs such as nurse‐managed online support groups [55], our qualitative findings revealed that participants desired more opportunities to connect online. Suggestions included links to platforms like Facebook to connect with other mothers, which aligns with evidence that online social support can provide accessible resources, shared experiences, and empowerment during the challenging post‐birth and early childhood period [56]. However, online platforms also carry potential drawbacks, including limited depth of engagement, overuse, and exposure to inappropriate content if not properly monitored [56]. Therefore, while C2u could consider incorporating and suggesting a greater variety of online platforms, it will be important to balance these benefits with strategies to mitigate potential negative effects.

Qualitative findings further revealed that mothers find it particularly challenging to maintain a good relationship with their partner when they have multiple children, which can affect both their perceived social support and parenting confidence. Prior research supports this, showing that having more children is a significant negative predictor of marital satisfaction [57]. Mothers also reported needing help with fostering connections between older children and a new sibling, underscoring the unique pressures faced by larger families. In light of these challenges, it may be valuable for C2u to refine its text messages to specifically support parents with multiple children, providing practical strategies and reminders for strengthening partner relationships and promoting sibling bonding. Future research could further examine which aspects of message content, frequency, or format are most effective in achieving these goals.

Notably, several mothers (regardless of number of children) emphasised the importance of self‐care. One participant described self‐care reminders as the most useful messages, while another noted that more concrete suggestions would be helpful, acknowledging that self‐care is ‘easier said than done’. Competing demands, such as caring for multiple children or balancing work and household responsibilities, often make self‐care difficult to practice [26]. Incorporating more structured and actionable self‐care guidance, such as step‐by‐step strategies, reflective prompts, or practical tips delivered via text, may help bridge the gap between intention and implementation. This aligns with evidence from a recent scoping review, which found that although digital platforms remain underutilised, they have significant potential to promote autonomy and support maternal well‐being, particularly when self‐care is challenging amidst competing demands [58].

A separate valuable finding of this study, which could be considered by Queensland Health as a whole, relates to the child health appointment booking system. Although 91.4% of participants agreed that C2u reminded them when their child's health check appointment was due, only 53% agreed that it made booking these appointments easier. Currently, the booking system does not support online bookings [59], meaning C2u messaging is limited to providing phone numbers for mothers to act upon. Globally, electronic booking systems are widely used and valued [60]. One study in Norway found that 80% of 2043 users found it easier and more efficient to book appointments online compared to by phone [61]. This could be an area for further exploration with young families in Queensland and considered by Queensland Health as a way to improve the efficiency and accessibility of the booking process.

Finally, overall satisfaction with C2u was high at T4. Participants expressed a desire for more messages and for the intervention to continue beyond their child's second birthday. This feedback has been implemented by CHQ HHS, with C2u messaging now extending until the child's fifth birthday [36]. The innovative approach of framing messages as though sent directly from the baby was also well received, as it gave the baby a voice before they could speak. A similar strategy is used in SMS4dads, an Australian text‐based intervention for antenatal and postpartum fathers, where participants reported that the ‘baby's voice’ messaging helped provide insight into the baby's mind and needs [62]. The continued use of this approach may be beneficial for other child health SMS interventions, and further research into this aspect of messaging could strengthen future findings.

### 4.1 Limitations

Despite its strengths as the first longitudinal evaluation of C2u, which provides an important evidence base for the program, several limitations should be noted. First, participants were not recruited using probability sampling, so the sample may not be fully representative of all C2u participants. As such, findings should be interpreted with caution. Additionally, we do not have basic demographic information for the broader C2u participant population, making it difficult to assess how representative our research sample was. Collecting these data in future evaluations could improve understanding of program users and enhance the generalisability of future findings.

Additionally, there was no control group, and parenting confidence can naturally improve as mothers gain experience [54]. Therefore, the extent to which observed improvements can be attributed to C2u remains uncertain. We also did not ask participants whether their partners were enrolled in C2u, which limited our ability to compare social support outcomes between those with and without partner involvement. Furthermore, it is unknown whether external contextual factors during the study period (e.g., COVID‐19 public health restrictions) may have influenced outcomes related to parental confidence or social support.

Finally, all outcomes were self‐reported, raising the possibility of social desirability or recall bias. Intervention engagement was not directly measured, making it unclear whether participants engaged consistently with all text messages, which could have influenced results. Moreover, qualitative findings may not fully capture the breadth of experiences across the broader population, potentially limiting the transferability of these insights.

## 5 Conclusion

This study introduced C2u as a supportive mHealth intervention for mothers and their children during the first 2 years. C2u appears to enhance maternal confidence, particularly among first‐time mothers, and to promote preventative health practices. Participants reported high satisfaction with the intervention, and these findings have informed its expansion to include antenatal care messaging and support for children up to 5 years of age.

Refining messaging on social support (including partner relationships, wider support networks, and sibling bonding) and self‐care may further strengthen outcomes, and future iterations should incorporate measures to assess these areas and evaluate their impact. Future research could also examine how parental education shapes engagement with mHealth interventions, including preferences for content and delivery. Importantly, a RCT, or an observational study designed to emulate a target trial, would provide robust evidence on the effectiveness of C2u. As the program continues to expand across Queensland, these findings provide a valuable foundation for informing future implementation and supporting broader efforts to enhance parenting support initiatives.

## Ethics Statement

The research protocol was approved by Children's Health Queensland Hospital and Health Service Human Research Ethics Committee (LNR/20/QCHQ/71980) and Griffith University Human Research Ethics Committee (2021/015).

## Conflicts of Interest

Authors from Centre for Children's Health and Wellbeing and Queensland Health contributed to the research in an independent capacity and were not involved in data collection or analysis to avoid potential bias. All authors declare that they have no additional conflicts of interest. This work was funded by the Australian Government, Department of Health and Aged Care, grant number DOH 4‐EFSPCZR.

## Supporting information


**Table S1:** Statements of support and satisfaction of C2u at T2.
**Table S2:** Statements of support and satisfaction of C2u at T3.

**Table S3:** Parenting confidence (0–6 months).
**Table S4:** Parenting confidence (6–12 months).
**Table S5:** Social Support (0–24 months).

## Data Availability

The data that support the findings of this study are available on request from the corresponding author. The data are not publicly available due to privacy or ethical restrictions.
