# Supplementary material for: Using mHealth to Support Queensland Mothers and Children From Birth to Two Years: A Longitudinal Study of Connecting2u
Source: Health Promot J Austr. 2025 Oct 14;36(4):e70117. doi: 10.1002/hpja.70117 (PMC12519932; doi:10.1002/hpja.70117)
Supplement: Supplementary file 1 — Table S1: Statements of support and satisfaction of C2u at T2. Table S2: Statements of support and satisfaction of C2u at T3. [file HPJA-36-0-s001.docx]

Table S1. Statements of support and satisfaction of C2u at T2 (n = 577)

|  | **Disagree** | | **Neither** | | **Agree** | |
| --- | --- | --- | --- | --- | --- | --- |
| **Statement** | **N** | **%** | **N** | **%** | **N** | **%** |
| **Support of relationships** |  |  |  |  |  |  |
| 1. The text messages helped my relationship with my partner/support person (n=471) | 71 | 12.3 | 245 | 42.5 | 155 | 26.9 |
| 1. Helped me feel emotionally supported when returning to daily activities e.g. returning to work | 51 | 8.8 | 269 | 46.6 | 257 | 44.5 |
| 1. I have shared the C2u information with others (e.g. partner, family, friends) | 128 | 22.2 | 89 | 15.4 | 360 | 62.4 |
| 1. Helped me feel closer to my child | 40 | 6.9 | 208 | 36.0 | 329 | 57.0 |
| 1. Increased my awareness of available social support groups in my area e.g. library playgroups | 94 | 16.3 | 160 | 27.7 | 323 | 56.0 |
| 1. Reminded me to take part in social support groups in my area such as mothers/parents’ groups and library playgroups | 38 | 6.6 | 149 | 25.8 | 390 | 67.7 |
| 1. Gave me ideas for interacting with my child (such as playing and reading with my child) | 21 | 3.6 | 64 | 11.1 | 492 | 85.3 |
| 1. The text messages were good for my relationship with my child | 13 | 2.3 | 131 | 22.7 | 433 | 75.0 |
| **Mothers’ wellbeing** |  |  |  |  |  |  |
| 1. Encouraged me to make healthier choices | 50 | 8.7 | 229 | 39.7 | 298 | 51.6 |
| 1. Made me feel more prepared for parenting than what I thought I would be without the text messages | 67 | 11.6 | 239 | 41.4 | 271 | 47.0 |
| 1. Reminded me to look after myself during these first 6 months of parenting | 22 | 3.8 | 66 | 11.4 | 489 | 84.7 |
| **Feeding practices** |  |  |  |  |  |  |
| 1. Gave me information about feeding practices that I have acted upon for my child | 71 | 12.3 | 152 | 26.3 | 354 | 61.4 |
| **Promotion of services** |  |  |  |  |  |  |
| 1. Made it easy to book in for the child health appointments | 81 | 14.0 | 272 | 47.1 | 224 | 38.8 |
| 1. The text messages reminded me to book my child for vaccinations | 48 | 8.3 | 121 | 21.0 | 408 | 70.7 |
| 1. Reminded me when my child’s health check appointment was due | 28 | 4.9 | 54 | 9.4 | 495 | 85.8 |
| **Satisfaction with C2u** |  |  |  |  |  |  |
| 1. The text messages helped me to feel supported | 15 | 2.6 | 90 | 15.6 | 472 | 81.8 |
| 1. I found the C2u text messages helpful | 12 | 2.1 | 62 | 10.7 | 503 | 87.2 |
| 1. I used the web links in the text messages to find more information | 85 | 14.7 | 125 | 21.7 | 367 | 63.6 |
| 1. I enjoyed being a part of C2u | 5 | 0.9 | 48 | 8.3 | 524 | 90.8 |
| 1. I would recommend C2u to other parents | 10 | 1.7 | 62 | 10.7 | 505 | 87.5 |
| 1. I understood all of the text messages | 2 | 0.3 | 3 | 0.5 | 572 | 99.1 |
| 1. I liked how many text messages I receive | 8 | 1.4 | 38 | 6.6 | 531 | 92.0 |
| 1. I was happy with the topics of the text messages | 2 | 0.3 | 21 | 3.6 | 554 | 96.0 |

Table S2. Statements of support and satisfaction of C2u at T3

|  | **Disagree** | | **Neither** | | **Agree** | |
| --- | --- | --- | --- | --- | --- | --- |
| **Statement** | **N** | **%** | **N** | **%** | **N** | **%** |
| **Support of relationships** |  |  |  |  |  |  |
| 1. The text messages helped my relationship with my partner/support person (n=288) | 80 | 27.8 | 138 | 47.9 | 70 | 24.3 |
| 1. Helped me feel emotionally supported when returning to daily activities e.g. returning to work (n=341) | 34 | 10 | 121 | 35.5 | 186 | 54.5 |
| 1. I have shared the C2u information with others (e.g. partner, family, friends) (n=345) | 61 | 17.7 | 49 | 14.2 | 235 | 68.1 |
| 1. Helped me feel closer to my child (n=341) | 25 | 7.3 | 116 | 34.0 | 200 | 58.7 |
| 1. Increased my awareness of available social support groups in my area e.g. library playgroups (n=341) | 38 | 11.1 | 81 | 23.8 | 222 | 65.1 |
| 1. Reminded me to take part in social support groups in my area such as mothers/parents’ groups and library playgroups (n=341) | 21 | 5.7 | 68 | 18.4 | 252 | 68.1 |
| 1. Gave me ideas for interacting with my child (such as playing and reading with my child) (n=341) | 14 | 4.1 | 33 | 9.7 | 294 | 86.2 |
| 1. The text messages were good for my relationship with my child (n=341) | 9 | 2.6 | 80 | 23.5 | 252 | 73.9 |
| **Mothers’ wellbeing (n=341)** |  |  |  |  |  |  |
| 1. Encouraged me to make healthier choices | 30 | 8.8 | 127 | 37.2 | 184 | 54.0 |
| 1. Made me feel more prepared for parenting than what I thought I would be without the text messages | 32 | 9.4 | 145 | 42.5 | 164 | 48.1 |
| 1. Reminded me to look after myself during these first 12 months of parenting | 4 | 1.2 | 31 | 9.1 | 306 | 89.7 |
| **Feeding practices (n=341)** |  |  |  |  |  |  |
| 1. Gave me information about feeding practices that I have acted upon for my child | 29 | 8.5 | 74 | 21.7 | 238 | 69.8 |
| **Promotion of services (n=341)** |  |  |  |  |  |  |
| 1. Made it easy to book in for the child health appointments | 41 | 12.0 | 150 | 44.0 | 150 | 44.0 |
| 1. The text messages reminded me to book my child for vaccinations | 21 | 6.2 | 55 | 16.1 | 265 | 77.7 |
| 1. Reminded me when my child’s health check appointment was due | 11 | 3.2 | 25 | 7.3 | 305 | 89.4 |
| **Satisfaction with C2u (n=345)** |  |  |  |  |  |  |
| 1. The text messages helped me to feel supported | 9 | 2.6 | 42 | 12.2 | 294 | 85.2 |
| 1. I found the C2u text messages helpful | 5 | 1.4 | 40 | 11.6 | 300 | 87.0 |
| 1. I used the web links in the text messages to find more information | 48 | 13.9 | 82 | 23.8 | 215 | 62.3 |
| 1. I enjoyed being a part of C2u | 1 | 0.3 | 25 | 7.2 | 319 | 92.5 |
| 1. I would recommend C2u to other parents | 166 | 48.1 | 30 | 8.7 | 149 | 43.2 |
| 1. I understood all of the text messages | 0 | 0 | 2 | 0.6 | 343 | 99.4 |
| 1. I liked how many text messages I receive | 1 | 0.3 | 17 | 4.9 | 327 | 94.8 |
| 1. I was happy with the topics of the text messages | 2 | 0.6 | 16 | 4.6 | 327 | 94.8 |
