# Supplementary material for: Using mHealth to Support Queensland Mothers and Children From Birth to Two Years: A Longitudinal Study of Connecting2u
Source: Health Promot J Austr. 2025 Oct 14;36(4):e70117. doi: 10.1002/hpja.70117 (PMC12519932; doi:10.1002/hpja.70117)
Supplement: Supplementary file 2 — Table S3: Parenting confidence (0–6 months). Table S4: Parenting confidence (6–12 months). Table S5: Social Support (0–24 months). [file HPJA-36-0-s002.docx]

| Table S3 – Parenting confidence (0-6months) | | | | | | | | | |
| --- | --- | --- | --- | --- | --- | --- | --- | --- | --- |
| **Model term*** | **Q25 Estimate (95% CI)** | **SE** | ***p*** | **Q50 Estimate (95% CI)** | **SE** | ***p*** | **Q75 Estimate (95% CI)** | **SE** | ***p*** |
| **Maternal age** |  |  |  |  |  |  |  |  |  |
| Intercept | 13.448 (11.351, 15.545) | 1.043 | <0.001 | 13.447 (11.349, 15.544) | 1.044 | <0.001 | 13.451 (11.354, 15.547) | 1.043 | <0.001 |
| Age | -0.105 (-0.193, -0.017) | 0.044 | 0.020 | -0.092 (-0.188, 0.004) | 0.048 | 0.059 | -0.052 (-0.103, -0.000) | 0.026 | 0.049 |
| Time (months) | 0.335 (0.002, 0.668) | 0.166 | 0.048 | 0.334 (0.002, 0.666) | 0.165 | 0.048 | 0.344 (0.013, 0.674) | 0.165 | 0.042 |
| Baseline confidence | 0.713 (0.620, 0.807) | 0.047 | 0.047 | 0.724 (0.627, 0.820) | 0.048 | <0.001 | 0.709 (0.645, 0.774) | 0.032 | <0.001 |
| Age x Time | 0.003 (-0.011, 0.017) | 0.007 | 0.642 | 0.007 (-0.008, 0.022) | 0.007 | 0.323 | 0.011 (-0.004, 0.026) | 0.007 | 0.163 |
| **Relationship** |  |  |  |  |  |  |  |  |  |
| Intercept | 13.168 (11.476, 14.861) | 0.842 | <0.001 | 13.169 (11.475, 14.862) | 0.843 | <0.001 | 13.169 (11.477, 14.861) | 0.842 | <0.001 |
| Relationship | 0.219 (-0.493, 0.931) | 0.354 | 0.539 | 0.219 (-0.493, 0.931) | 0.354 | 0.539 | 0.219 (-0.493, 0.931) | 0.354 | 0.539 |
| Time (months) | 0.387 (0.313, 0.461) | 0.037 | <0.001 | 0.387 (0.313, 0.462) | 0.037 | <0.001 | 0.394 (0.311, 0.477) | 0.042 | <0.001 |
| Baseline confidence | 0.623 (0.581, 0.665) | 0.021 | <0.001 | 0.653 (0.610, 0.697) | 0.022 | <0.001 | 0.674 (0.631, 0.718) | 0.022 | <0.001 |
| Relationship x Time | 0.081 (-0.239, 0.401) | 0.159 | 0.613 | 0.082 (-0.239, 0.402) | 0.159 | 0.611 | 0.082 (-0.237, 0.401) | 0.159 | 0.606 |
| **First child** |  |  |  |  |  |  |  |  |  |
| Intercept | 14.020 (12.348, 15.691) | 0.832 | <0.001 | 14.039 (12.364, 15.714) | 0.833 | <0.001 | 14.040 (12.368, 15.711) | 0.832 | <0.001 |
| First child | -0.414 (-0.743, -0.084) | 0.164 | 0.015 | -0.394 (-0.729, -0.059) | 0.167 | 0.022 | -0.395 (-0.725, -0.064) | 0.164 | 0.020 |
| Time (months) | 0.167 (0.045, 0.290) | 0.061 | 0.009 | 0.202 (0.074, 0.330) | 0.064 | 0.003 | 0.221 (0.080, 0.363) | 0.070 | 0.003 |
| Baseline confidence | 0.609 (0.562, 0.656) | 0.024 | <0.001 | 0.641 (0.601, 0.681) | 0.020 | <0.001 | 0.664 (0.628, 0.701) | 0.018 | <0.001 |
| First child x Time | 0.240 (0.082, 0.399) | 0.079 | 0.004 | 0.250 (0.109, 0.391) | 0.070 | 0.001 | 0.261 (0.115, 0.407) | 0.073 | 0.001 |
| **Education** |  |  |  |  |  |  |  |  |  |
| Intercept | 13.446 (11.686, 15.207) | 0.876 | <0.001 | 13.447 (11.682, 15.212) | 0.878 | <0.001 | 13.440 (11.680, 15.200) | 0.876 | <0.001 |
| Diploma or trade | -0.283 (-0.697, 0.130) | 0.206 | 0.175 | -0.283 (-0.696, 0.130) | 0.206 | 0.175 | -0.285 (-0.698, 0.129) | 0.206 | 0.173 |
| University | -0.336 (-0.799, 0.128) | 0.231 | 0.152 | -0.335 (-0.799, 0.130) | 0.231 | 0.154 | -0.339 (-0.801, 0.124) | 0.230 | 0.147 |
| Time (months) | 0.294 (0.019, 0.569) | 0.137 | 0.036 | 0.294 (0.030, 0.559) | 0.132 | 0.030 | 0.315 (0.043, 0.586) | 0.135 | 0.024 |
| Baseline confidence | 0.625 (0.579, 0.670) | 0.023 | <0.001 | 0.655 (0.611, 0.698) | 0.022 | <0.001 | 0.677 (0.633, 0.721) | 0.022 | <0.001 |
| Diploma or trade x Time | 0.061 (-0.229, 0.350) | 0.144 | 0.675 | 0.060 (-0.232, 0.353) | 0.146 | 0.680 | 0.062 (-0.227, 0.350) | 0.144 | 0.669 |
| University x Time | 0.118 (-0.173, 0.410 | 0.145 | 0.419 | 0.119 (-0.178, 0.417) | 0.148 | 0.424 | 0.140 (-0.155, 0.435) | 0.147 | 0.346 |
| **Income** |  |  |  |  |  |  |  |  |  |
| Intercept | 13.178 (11.446, 14.910) | 0.862 | <0.001 | 13.189 (11.461, 14.918) | 0.860 | <0.001 | 13.188 (11.452, 14.924) | 0.864 | <0.001 |
| 80–120K | 0.014 (-0.400, 0.428) | 0.206 | 0.946 | 0.012 (-0.398, 0.423) | 0.204 | 0.952 | 0.012 (-0.392, 0.416) | 0.201 | 0.954 |
| >120K | 0.106 (-0.333, 0.545) | 0.219 | 0.630 | 0.124 (-0.314, 0.562) | 0.218 | 0.572 | 0.123 (-0.318, 0.563) | 0.219 | 0.577 |
| Time (months) | 0.231 (0.027, 0.436) | 0.102 | 0.027 | 0.238 (0.037, 0.440) | 0.100 | 0.021 | 0.246 (0.035, 0.458) | 0.105 | 0.023 |
| Baseline confidence | 0.634 (0.587, 0.680) | 0.023 | <0.001 | 0.650 (0.605, 0.694) | 0.022 | <0.001 | 0.674 (0.630, 0.718) | 0.022 | <0.001 |
| 80–120K x Time | 0.131 (-0.057, 0.320) | 0.094 | 0.168 | 0.155 (-0.051, 0.361) | 0.103 | 0.138 | 0.154 (-0.054, 0.362) | 0.104 | 0.143 |
| >120K x Time | 0.163 (-0.048, 0.375) | 0.105 | 0.127 | 0.170 (-0.035, 0.376) | 0.102 | 0.101 | 0.172 (-0.040, 0.384) | 0.105 | 0.109 |

**Reference groups: Relationship = married/partnered, First Child (Parity)= not first child, Education = did not complete high school/completed high school, Income = <80,000. Coding for all covariates as described in Methods of main manuscript.*

| Table S4 – Parenting confidence (6-12months) | | | | | | | | | |
| --- | --- | --- | --- | --- | --- | --- | --- | --- | --- |
| **Model term*** | **Q25 Estimate (95% CI)** | **SE** | ***p*** | **Q50 Estimate (95% CI)** | **SE** | ***p*** | **Q75 Estimate (95% CI)** | **SE** | ***p*** |
| **Maternal age** |  |  |  |  |  |  |  |  |  |
| Intercept | 4.528 (1.273, 7.783) | 1.620 | 0.007 | 4.528 (1.273, 7.783) | 1.620 | 0.007 | 4.528 (1.273, 7.783) | 1.620 | 0.007 |
| Age | 0.002 (-0.084, 0.087) | 0.043 | 0.969 | 0.008 (-0.077, 0.093) | 0.042 | 0.852 | 0.006 (-0.079, 0.091) | 0.042 | 0.886 |
| Time (months) | 0.128 (-0.327, 0.583) | 0.226 | 0.574 | 0.128 (-0.327, 0.583) | 0.226 | 0.573 | 0.129 (-0.326, 0.584) | 0.226 | 0.572 |
| 6 mo Baseline confidence | 0.864 (0.821, 0.907) | 0.021 | <0.001 | 0.874 (0.830, 0.918) | 0.022 | <0.001 | 0.872 (0.828, 0.916) | 0.022 | <0.001 |
| Age x Time | -0.004 (-0.017, 0.010) | 0.007 | 0.605 | -0.001 (-0.015, 0.012) | 0.007 | 0.846 | 0.000 (-0.013, 0.013) | 0.007 | 0.993 |
| **Relationship** |  |  |  |  |  |  |  |  |  |
| Intercept | 4.610 (3.145, 6.074) | 0.729 | <0.001 | 4.610 (3.146, 6.075) | 0.729 | <0.001 | 4.611 (3.146, 6.076) | 0.729 | <0.001 |
| Relationship | 0.804 (-0.835, 2.443) | 0.815 | 0.329 | 0.804 (-0.835, 2.442) | 0.815 | 0.329 | 0.804 (-0.835, 2.443) | 0.815 | 0.329 |
| Time (months) | 0.082 (0.011, 0.153) | 0.035 | 0.024 | 0.095 (0.038, 0.151) | 0.028 | 0.002 | 0.099 (0.040, 0.159) | 0.030 | 0.002 |
| 6 mo Baseline confidence | 0.847 (0.810, 0.885) | 0.019 | <0.001 | 0.877 (0.841, 0.912) | 0.018 | <0.001 | 0.879 (0.835, 0.923) | 0.022 | <0.001 |
| Relationship x Time | -0.109 (-0.369, 0.150) | 0.129 | 0.401 | -0.110 (-0.369, 0.149) | 0.129 | 0.399 | -0.109 (-0.368, 0.150) | 0.129 | 0.402 |
| **First child** |  |  |  |  |  |  |  |  |  |
| Intercept | 4.343 (2.989, 5.696) | 0.673 | <0.001 | 4.344 (2.990, 5.697) | 0.673 | <0.001 | 4.344 (2.990, 5.697) | 0.673 | <0.001 |
| First child | -0.923 (-1.616, -0.229) | 0.345 | 0.010 | -0.921 (-1.615, -0.228) | 0.345 | 0.010 | -0.921 (-1.615, -0.227) | 0.345 | 0.010 |
| Time (months) | -0.046 (-0.145, 0.052) | 0.049 | 0.348 | -0.027 (-0.117, 0.063) | 0.045 | 0.546 | -0.024 (-0.109, 0.062) | 0.042 | 0.582 |
| 6 mo Baseline confidence | 0.877 (0.837, 0.917) | 0.020 | <0.001 | 0.899 (0.866, 0.933) | 0.017 | <0.001 | 0.902 (0.862, 0.942) | 0.020 | <0.001 |
| First child x Time | 0.147 (0.033, 0.260) | 0.056 | 0.012 | 0.162 (0.050, 0.275) | 0.056 | 0.006 | 0.165 (0.051, 0.278) | 0.056 | 0.005 |
| **Education** |  |  |  |  |  |  |  |  |  |
| Intercept | 5.448 (3.867, 7.028) | 0.787 | <0.001 | 5.449 (3.868, 7.029) | 0.787 | <0.001 | 5.449 (3.869, 7.030) | 0.786 | <0.001 |
| Diploma or trade | -0.513 (-1.578, 0.552) | 0.530 | 0.338 | -0.513 (-1.578, 0.552) | 0.530 | 0.338 | -0.513 (-1.578, 0.552) | 0.530 | 0.338 |
| University | -0.920 (-1.816, -0.024) | 0.446 | 0.044 | -0.919 (-1.815, -0.023) | 0.446 | 0.045 | -0.918 (-1.814, -0.022) | 0.446 | 0.045 |
| Time (months) | -0.060 (-0.202, 0.081) | 0.070 | 0.396 | -0.031 (-0.172, 0.109) | 0.070 | 0.657 | -0.024 (-0.164, 0.115) | 0.069 | 0.727 |
| 6 mo Baseline confidence | 0.854 (0.819, 0.888) | 0.017 | <0.001 | 0.875 (0.841, 0.910) | 0.017 | <0.001 | 0.902 (0.853, 0.952) | 0.025 | <0.001 |
| Diploma or trade x Time | 0.070 (-0.097, 0.238) | 0.083 | 0.403 | 0.076 (-0.093, 0.245) | 0.084 | 0.372 | 0.075 (-0.093, 0.243) | 0.084 | 0.373 |
| University x Time | 0.124 (-0.016, 0.265) | 0.070 | 0.081 | 0.149 (0.009, 0.288) | 0.070 | 0.038 | 0.155 (0.016, 0.295) | 0.069 | 0.030 |
| **Income** |  |  |  |  |  |  |  |  |  |
| Intercept | 4.877 (3.410, 6.345) | 0.730 | <0.001 | 4.878 (3.410, 6.346) | 0.730 | <0.001 | 4.878 (3.410, 6.345) | 0.730 | <0.001 |
| 80–120K | -0.160 (-1.042, 0.722) | 0.439 | 0.717 | -0.160 (-1.042, 0.722) | 0.439 | 0.717 | -0.159 (-1.041, 0.722) | 0.439 | 0.718 |
| >120K | -0.298 (-1.166, 0.571) | 0.432 | 0.494 | -0.297 (-1.165, 0.571) | 0.432 | 0.496 | -0.298 (-1.166, 0.570) | 0.432 | 0.494 |
| Time (months) | 0.014 (-0.128, 0.155) | 0.071 | 0.849 | 0.032 (-0.112, 0.175) | 0.071 | 0.660 | 0.054 (-0.089, 0.196) | 0.071 | 0.453 |
| 6 mo Baseline confidence | 0.852 (0.812, 0.892) | 0.020 | <0.001 | 0.875 (0.840, 0.910) | 0.017 | <0.001 | 0.875 (0.828, 0.923) | 0.024 | <0.001 |
| 80–120K x Time | 0.044 (-0.100, 0.188) | 0.072 | 0.539 | 0.048 (-0.096, 0.192) | 0.072 | 0.506 | 0.066 (-0.077, 0.209) | 0.071 | 0.359 |
| >120K x Time | 0.064 (-0.078, 0.206) | 0.070 | 0.370 | 0.079 (-0.062, 0.221) | 0.070 | 0.264 | 0.076 (-0.066, 0.217) | 0.070 | 0.288 |

**Reference groups: Relationship = married/partnered, First Child (Parity)= not first child, Education = did not complete high school/completed high school, Income = <80,000. Coding for all covariates as described in Methods of main manuscript.*

| Table S5 – Social Support (0–24 months) | | | | | | | | | |
| --- | --- | --- | --- | --- | --- | --- | --- | --- | --- |
| **Model term*** | **Q25 Estimate (95% CI)** | **SE** | ***p*** | **Q50 Estimate (95% CI)** | **SE** | ***p*** | **Q75 Estimate (95% CI)** | **SE** | ***p*** |
| **Maternal age** |  |  |  |  |  |  |  |  |  |
| Intercept | 16.283 (10.934, 21.631) | 2.662 | <0.001 | 16.285 (10.939, 21.631) | 2.660 | <0.001 | 16.299 (10.951, 21.648) | 2.662 | 2.662 |
| Age | -0.382 (-0.538, -0.227) | 0.078 | <0.001 | -0.333 (-0.479, -0.186) | 0.073 | <0.001 | -0.091 (-0.232, 0.051) | 0.070 | 0.204 |
| Time (months) | 0.232 (-0.232, 0.695) | 0.231 | 0.320 | 0.242 (-0.213, 0.696) | 0.226 | 0.291 | 0.269 (-0.190, 0.729) | 0.228 | 0.244 |
| Baseline social support | 0.923 (0.844, 1.002) | 0.039 | <0.001 | 0.932 (0.888, 0.976) | 0.022 | <0.001 | 0.860 (0.799, 0.920) | 0.030 | <0.001 |
| Age x Time | -0.012 (-0.032, 0.008) | 0.01 | 0.228 | -0.008 (-0.021, 0.006) | 0.007 | 0.274 | -0.007 (-0.029, 0.014) | 0.011 | 0.51 |
| **Relationship** |  |  |  |  |  |  |  |  |  |
| Intercept | 14.701 (10.601, 18.800) | 2.040 | <0.001 | 14.750 (10.646, 18.854) | 2.042 | <0.001 | 14.752 (10.649, 18.855) | 2.042 | <0.001 |
| Relationship | 0.689 (-2.599, 3.978) | 1.636 | 0.676 | 0.692 (-2.594, 3.978) | 1.635 | 0.674 | 0.692 (-2.594, 3.979) | 1.635 | 0.674 |
| Time (months) | -0.041 (-0.162, 0.079) | 0.060 | 0.493 | 0.046 (-0.070, 0.163) | 0.058 | 0.425 | 0.056 (-0.041, 0.154) | 0.049 | 0.253 |
| Baseline social support | 0.764 (0.701, 0.827) | 0.031 | <0.001 | 0.818 (0.768, 0.869) | 0.025 | <0.001 | 0.846 (0.800, 0.892) | 0.023 | <0.001 |
| Relationship x Time | 0.013 (-0.382, 0.408) | 0.197 | 0.947 | -0.022 (-0.428, 0.384) | 0.202 | 0.913 | -0.022 (-0.441, 0.397) | 0.209 | 0.915 |
| **First child** |  |  |  |  |  |  |  |  |  |
| Intercept | 13.242 (9.862, 16.622) | 1.682 | <0.001 | 13.322 (9.951, 16.692) | 1.677 | <0.001 | 13.349 (9.974, 16.725) | 1.680 | <0.001 |
| First child | 1.240 (-0.286, 2.765) | 0.759 | 0.109 | 1.263 (-0.251, 2.777) | 0.754 | 0.100 | 1.281 (-0.238, 2.800) | 0.756 | 0.096 |
| Time (months) | -0.338 (-0.618, -0.058) | 0.139 | 0.019 | 0.072 (-0.164, 0.309) | 0.118 | 0.541 | 0.119 (-0.125, 0.362) | 0.121 | 0.332 |
| Baseline social support | 0.782 (0.728, 0.836) | 0.027 | <0.001 | 0.826 (0.782, 0.871) | 0.022 | <0.001 | 0.854 (0.815, 0.892) | 0.019 | <0.001 |
| First child x Time | -0.280 (-0.578, 0.018) | 0.148 | 0.065 | -0.139 (-0.410, 0.132) | 0.135 | 0.308 | -0.112 (-0.367, 0.144) | 0.127 | 0.384 |
| **Education** |  |  |  |  |  |  |  |  |  |
| Intercept | 12.954 (9.168, 16.741) | 1.884 | <0.001 | 13.022 (9.218, 16.825) | 1.893 | <0.001 | 13.085 (9.276, 16.894) | 1.895 | <0.001 |
| Diploma or trade | 1.267 (-1.183, 3.716) | 1.219 | 0.304 | 1.291 (-1.141, 3.723) | 1.210 | 0.291 | 1.306 (-1.126, 3.738) | 1.210 | 0.286 |
| University | 1.905 (-0.078, 3.888) | 0.987 | 0.059 | 1.933 (0.067, 3.800) | 0.929 | 0.043 | 1.986 (0.119, 3.853) | 0.929 | 0.038 |
| Time (months) | 0.187 (-0.126, 0.500) | 0.156 | 0.236 | 0.227 (-0.015, 0.470) | 0.121 | 0.066 | 0.268 (0.036, 0.500) | 0.115 | 0.024 |
| Baseline social support | 0.772 (0.710, 0.834) | 0.031 | <0.001 | 0.819 (0.772, 0.867) | 0.024 | <0.001 | 0.846 (0.806, 0.887) | 0.020 | <0.001 |
| Diploma or trade x Time | -0.295 (-0.607, 0.017) | 0.155 | 0.063 | -0.282 (-0.558, -0.007) | 0.137 | 0.045 | -0.215 (-0.489, 0.060) | 0.137 | 0.123 |
| University x Time | -0.258 (-0.577, 0.061) | 0.159 | 0.110 | -0.228 (-0.467, 0.012) | 0.119 | 0.062 | -0.245 (-0.501, 0.011) | 0.127 | 0.061 |
| **Income** |  |  |  |  |  |  |  |  |  |
| Intercept | 13.482 (8.989, 17.975) | 2.236 | <0.001 | 13.575 (9.064, 18.086) | 2.245 | <0.001 | 13.585 (9.068, 18.102) | 2.248 | <0.001 |
| 80–120K | 1.057 (-0.627, 2.740) | 0.838 | 0.213 | 1.080 (-0.606, 2.766) | 0.839 | 0.204 | 1.082 (-0.606, 2.769) | 0.840 | 0.204 |
| >120K | 2.224 (0.561, 3.887) | 0.828 | 0.010 | 2.268 (0.596, 3.941) | 0.832 | 0.009 | 2.277 (0.612, 3.942) | 0.828 | 0.008 |
| Time (months) | -0.345 (-0.698, 0.008) | 0.176 | 0.055 | -0.136 (-0.395, 0.124) | 0.129 | 0.298 | -0.108 (-0.357, 0.142) | 0.124 | 0.391 |
| Baseline social support | 0.761 (0.698, 0.823) | 0.031 | <0.001 | 0.812 (0.761, 0.864) | 0.026 | <0.001 | 0.841 (0.797, 0.886) | 0.022 | <0.001 |
| 80–120K x Time | 0.089 (-0.160, 0.338) | 0.124 | 0.476 | 0.212 (-0.020, 0.444) | 0.115 | 0.072 | 0.240 (-0.002, 0.482) | 0.120 | 0.052 |
| >120K x Time | 0.148 (-0.126, 0.422) | 0.136 | 0.283 | 0.135 (-0.113, 0.384) | 0.124 | 0.280 | 0.159 (-0.095, 0.412) | 0.126 | 0.214 |

**Reference groups: Relationship = married/partnered, First Child (Parity) = not first child, Education = did not complete high school/completed high school, Income = <80,000. Coding for all covariates as described in Methods of main manuscript.*
